# Neurotized Free Muscle Flaps can Produce MRI Changes Mimicking Tumour Recurrence

**DOI:** 10.1080/13577140500340484

**Published:** 2005

**Authors:** J. K. O’Neill, J. A. Barrett, T. Cobley, V. Devaraj, D. A. T. Silver

**Affiliations:** Departments of Radiology and Plastic and Reconstructive Surgery Royal Devon and Exeter Hospital Barrack Road Exeter EX2 5DW UK

## Abstract

Soft tissue sarcomas are investigated by magnetic resonance imaging (MRI) both for initial staging and follow-up. We describe the presence of increased signal on T2-weighted images caused by a neurotized muscle flap following reconstructive surgery. This raised concern about possible sarcoma recurrence that was not clinically evident. On post-operative imaging of sarcomas the presence of recurrent tumour is indicated by a mass and high signal intensity on T2-weighted images. However, high signal changes in skeletal muscle on T2-weighted images are not specific. In this case, the free functioning muscle transfer with neurotization of the flap mimicked recurrence on MR scan. High signal intensity on T2-weighted images in muscle is an indication of either a physiological change or a pathological condition and must be taken in context of the clinical picture.


*Sarcoma*, September/December 2005; 9(3/4): 133–136

Taylor & Francis

Taylor & Francis Group

**ORIGINAL ARTICLE**

### Neurotized free muscle flaps can produce MRI changes mimicking tumour recurrence

J. K. O'NEILL, J. A. BARRETT, T. COBLEY, V. DEVARAJ, & D. A. T. SILVER

*Departments of Radiology and Plastic and Reconstructive Surgery, Royal Devon and Exeter Hospital, Barrack Road, Exeter EX2 5DW, UK*

**Abstract**
Soft tissue sarcomas are investigated by magnetic resonance imaging (MRI) both for initial staging and follow-up. We describe the presence of increased signal on T2-weighted images caused by a neurotized muscle flap following reconstructive surgery. This raised concern about possible sarcoma recurrence that was not clinically evident. On post-operative imaging of sarcomas the presence of recurrent tumour is indicated by a mass and high signal intensity on T2-weighted images. However, high signal changes in skeletal muscle on T2-weighted images are not specific. In this case, the free functioning muscle transfer with neurotization of the flap mimicked recurrence on MR scan. High signal intensity on T2-weighted images in muscle is an indication of either a physiological change or a pathological condition and must be taken in context of the clinical picture.

**Keywords:** *Magnetic resonance, sarcoma, recurrence, neurotized, flap*

### Introduction

Magnetic resonance imaging (MRI) is the primary imaging modality for lesion detection, local staging and follow-up of soft tissue sarcomas. This is because of the pluridirectional imaging capability and good contrast resolution that MRI provides [1–5].

Sarcoma shows a mass effect and a high water content relative to the surrounding tissues and therefore a T2-weighted or short tau inversion recovery (STIR) sequence will predominantly demonstrate a high signal intensity mass. A T1-weighted image of a sarcoma will be low signal [5,6]. Tumours usually enhance with gadolonium chelate contrast.

Radiological difficulties in assessing for recurrent soft tissue sarcoma may be encountered for a number of reasons. After surgery the normal anatomy may be distorted and the disposition of muscles may be deliberately altered at surgery making imaging more difficult to interpret [6].

It is not always the case that gadolinium chelate contrast causes a tumour mass to enhance and, furthermore, conditions such as inflammation cause enhancement too. As an exception to the rule, some types of sarcomas have low signal intensity on T2-weighted MR images. In addition, there are postoperative conditions other than recurrence that can cause high intensity signal on T2-weighted MR images [6,7].

We describe the presence of increased signal on T2-weighted images caused by a neurotized muscle flap following reconstructive surgery. This raised concern about possible sarcoma recurrence that was not clinically evident.

### *Case report*

A 54-year-old right-handed male artist initially presented with a mass on the extensor surface of his left forearm. Histological analysis of an excision biopsy revealed a probable leiomyosarcoma. Following local recurrence the patient underwent wide local excision with a split skin graft and radiotherapy that was completed 9 months after his original presentation. Further histology confirmed complete local excision of a high grade spindle cell sarcoma with myogenic differentiation.

Correspondence: Miss Jennifer O'Neill, Plastic and Reconstructive Surgery Department, Royal Devon and Exeter Hospital (Wonford), Barrack Road, EX2 5DW, UK. Tel: 44 1392 411611. Fax: 44 1392 402894. E-mail: jenniferoneill@hotmail.com

ISSN 1357–714X print/ISSN 1369–1643 online/05/(03-04)0133-4 © 2005 Taylor & Francis
DOI: 10.1080/13577140500340484

An area of radionecrosis and ulceration was excised and resurfaced with a pedicled radial forearm flap. There was no histological recurrence.

Three and a half years after his initial presentation further disease was evident and he was seen at our unit. He underwent excision of the extensor compartment of the forearm and resurfacing with a free functioning muscle transfer of the latissimus dorsi with neurotization of the thoracodorsal nerve using the posterior interosseous nerve as a donor. At this time the histology reported a high grade spindle cell sarcoma/leiomyosarcoma with complete excision. Further radiotherapy was precluded given the high dose of radiation the patient had already received. At that time, staging investigations revealed no evidence of distant disease and the patient made an uneventful recovery. He then commenced galvanic muscle stimulation.

Six months after the surgery, MR imaging was performed. This showed normal signal and morphological characteristics of the muscle within the forearm on T1-weighted images. However, on the T2-weighted images there was a diffuse increase in signal throughout the extensor compartment (see Figures 1–4). At first, concern about further recurrence was raised although there was no mass palpable.

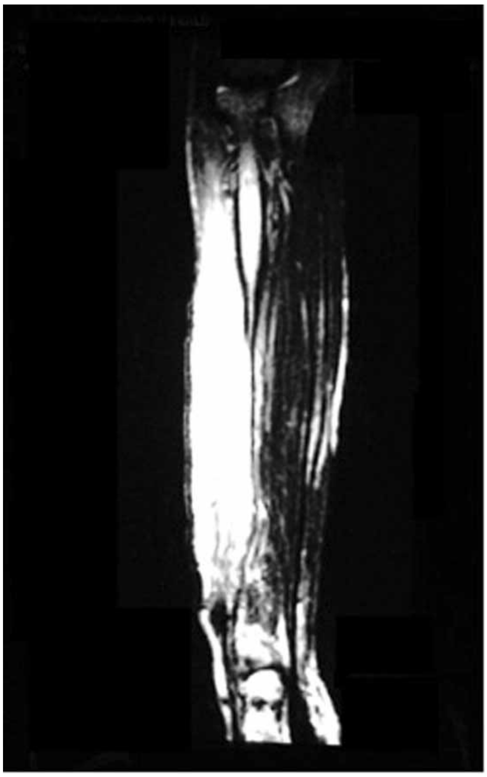

Figure 1. Coronal T2-weighted scan showing extensive high signal within the muscle flap – the abnormal signal extends throughout the entire length of the flap. This distribution mitigates against this representing a recurrent tumour.

### Discussion

The soft tissue contrast obtained by MR imaging is superior to that obtained by ultrasound or computed tomography [4,6]. Even though there may be radiological difficulties, it is a most valuable tool in monitoring for recurrence of soft tissue sarcomas. Its use in evaluating spread into adjacent compartments and bone or vascular invasion is well described [4] and this is facilitated by the ability to image in multiple planes [2,3,5,6]. On post-operative imaging the presence of recurrent tumour is indicated by a mass and high signal intensity on T2-weighted images.

Differentiation between recurrent tumour and fluid collections can be facilitated by the use of paramagnetic contrast agents [6,8], a tumour mass will enhance but a seroma will show no more than minor rim enhancement.

However, there are possible pitfalls with application of these rules. Contrast enhancement similar to that seen with a recurrent tumour can also occur with inflammation or infection or the rare radiation-induced pseudomass [6,8]. Furthermore, if the tumour is hypovascular (e.g., lobules of cartilage in recurrent chondrosarcoma) there will be only rim enhancement with contrast and it may be mistaken for a seroma [6].

In the absence of a high signal intensity mass the likelihood of recurrence is remote [6]. However, it is possible for a tumour to give a low signal intensity on the T2-weighted image if it is densely mineralized (e.g., osteosarcoma) or hypocellular (e.g., fibromatosis) [6].

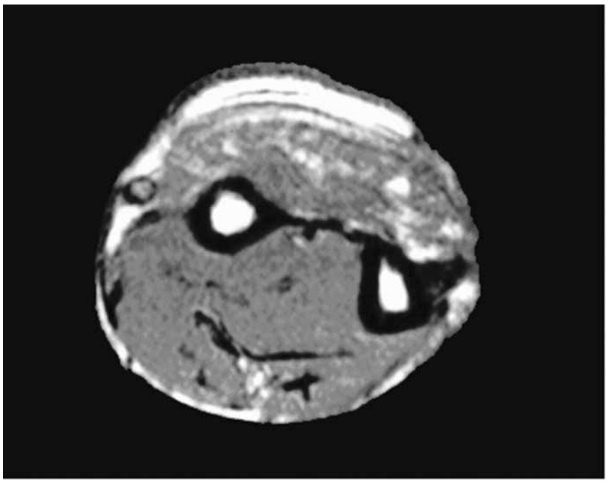

Figure 2. Axial T1 image showing some high signal area, representing fatty atrophy, within the extensor compartment that has been resurfaced with the neurotized free tissue transfer.

In addition to these difficulties, high signal changes in skeletal muscle on T2-weighted images are not specific and have been described in circumstances such as post exercise or strength training and in pathological conditions including inflammatory myopathies, rhabdomyolysis, hypokalaemic

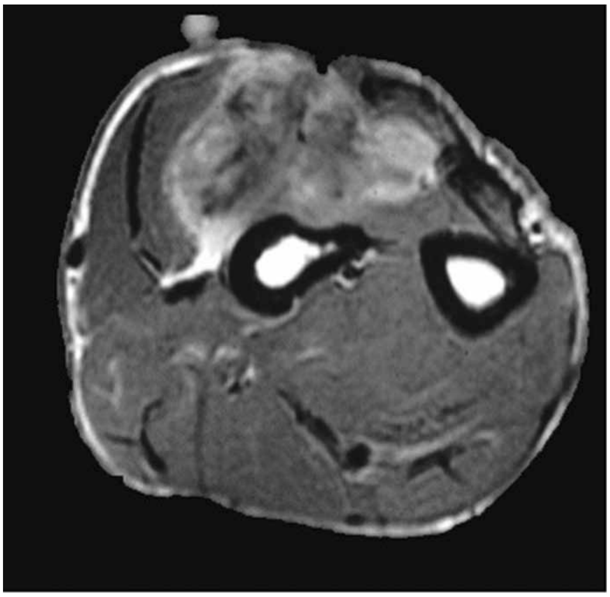

Figure 3. Axial T1 post-gadolinium enhancement showing areas of increased signal. Although this is not specific, it can occur as a result of neovascularisation and possibly neurotization.

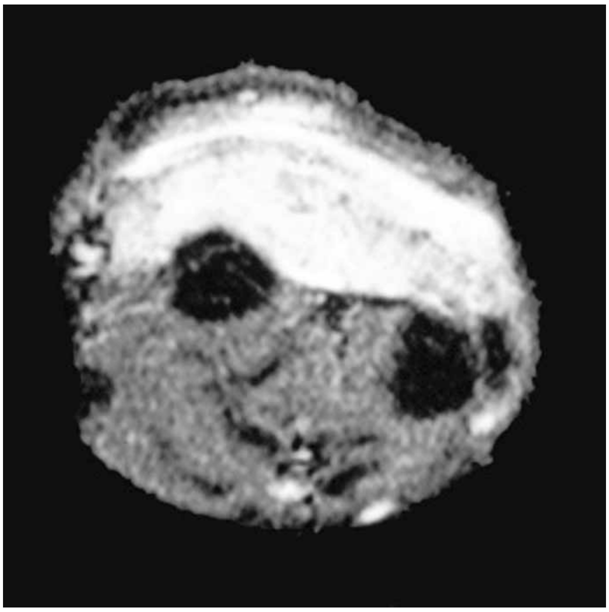

Figure 4. STIR T1-weighted fat suppressed image showing marked high signal representative of oedema within the flap.

periodic paralysis, degenerative myopathies, sport-related injuries, compartment syndrome, infection with abscess formation and acute and subacute denervation [6,9–12].

High signal changes are also caused by many postoperative conditions and complications. Panicek et al. [7] reviewed 39 MRI examinations of 11 patients who had undergone treatment for malignant musculoskeletal neoplasm. The patient's examinations were selected from an 'interesting case' file where they had been filed because of high signal intensity on T2-weighted MR images that could be indicative of a recurrent or residual neoplasm. They reviewed the clinical history of each patient and none of the 11 patients had true recurrent sarcoma. In their study they found that the non-neoplastic entities responsible for high signal intensity areas included post-radiation therapy changes, fat necrosis, surgical haemostatic packing material, intercalary bone allograft, strut bone graft, atrophic muscle, haematomas, seromas and herniated colon and bladder. The authors did not examine all MR images performed at their institution in the time interval that these cases presented so, unfortunately, the prevalence of the findings could not be determined.

Information on the post-operative course of the patient is vital when interpreting MRI studies and knowledge of details of the surgical procedure and the interval since surgery or irradiation will aid accurate interpretation of the MR imaging findings.

Richardson et al. [13] studied the time course, distribution and degree of post-radiation oedema leading to signal changes on MR imaging in 15 patients who underwent radiotherapy after resection of musculoskeletal sarcomas. Their method involved serial MR studies on each patient to monitor the size and signal intensity of the subcutaneous fat, muscle and intramuscular septa/fascial planes. They found that there is a relatively wide variation but the oedema seems to persist longer in the intramuscular septa than fat or muscle. Although the duration of follow-up was limited in their study, the results showed that this oedema is sometimes still present 3–4 years after treatment.

In this case, the last dose of radiotherapy was 3 years and 3 months prior to these MR images. Post-radiation oedema that could be responsible for increased signal intensity on the T2-weighted image was likely to have resolved but might have still been present.

Denervation of a muscle, for example as a result of surgery, leads to predictable changes in MR signal characteristics [12]. In the acute stage no abnormality may be seen but after 2–4 weeks (subacute phase) there is an increase in T2 signal intensity as a result of muscle 'oedema' and the muscle appears enlarged [6]. This is thought to be due to the shift of water from intracellular to extracellular spaces [11]. In the chronic phase fatty atropy of the muscle takes place and is visible on the MRI [6,14].

The finding of fat within the lesion in this case presents the radiological differential diagnosis of other lesions that contain fat such as haemangiomas and some histological subtypes of liposarcoma. The fat also gives the skeletal muscle a 'textural pattern', on high resolution T1-weighted spin echo images, which may indicate the benign nature of the lesion. Biondetti and Ehman [15] examined the MR imaging studies of 40 patients who had previously undergone surgery and radiation therapy for soft tissue sarcomas of the lower extremities. They found that 31 of the patients' images showed T2-weighted high signal intensity and, of those, 23 had the 'texture sign' on T1-weighted images. None of these patients proved to have tumour recurrence. However, out of the eight who had high signal on T2-weighted images and no texture sign there were two cases of tumour recurrence.

In this case, the free functioning muscle transfer with neurotization of the flap mimicked recurrence on MR scan. The appearance of an almost uniform high signal throughout the flap is in keeping with an abnormal state of muscle excitement, which is thought to be due to the galvanic stimulation, causing increased muscle activity and new neural stimulation. The increased signal may arise from neurotization with an abnormal state of excitement.

High signal intensity on T2-weighted MR images in muscle is an indication of either a physiological change or a pathological condition and must be taken in context of the clinical picture.

### References

1. Borden EC, Baker LH, Bell RS, Bramwell V, Demetri GD, Eisenburg BL, Fletcher CD, Fletcher JA, Ladanyi M, Meltzer P, et al. Soft tissue sarcomas of adults: State of the translational science. Clin Cancer Res 2003;9:1941–1956.
2. Sundarum M, McQuire MH, Herbold DR. Magnetic resonance imaging of soft tissue masses: An evaluation of 53 histological proven tumours. Magn Reson Imaging 1988;6:237–248.
3. Kransdorf MJ, Jelinek JS, Moser RP, Utz JA, Brower AC, Hudson TM, Berrey BH. Soft tissue masses: Diagnosis using MR imaging. Am J Radiol 1989;153:541–547.
4. Pang KK, Hughes T. MR imaging of the musculoskeletal soft tissue mass: Is heterogeneity a sign of malignancy? J Clin Med Assoc 2003;66(11):655–661.
5. Hanna SL, Fletcher BD. MR imaging of malignant soft-tissue tumors. Mag Reson Imaging Clin N Am 1995; 3(4):629–650.
6. Davies AM, Vanel D. Follow-up of musculoskeletal tumors. I. Local Recurrence. Eur Radiol 1998;8:791–799.
7. Panicek DM, Schwartz LH, Heelan JF, Caravelli JF. Non-neoplastic causes of high signal intensity at T2-weighted MR imaging after treatment for musculoskeletal neoplasm. Skeletal Radiol 1995;24:185–190.
8. Vanel D, Shapeeero LG, De Baere T, Gilles R, Tardivon A, Genin J, Guinebreti. MR imaging in the follow-up of malignant and aggressive soft-tissue tumours: Results of 511 examinations. Radiology 1994;190:263–268.
9. Jehenson P, Leroy-Willig A, De Kerviler E, Duboc D, Syrota A. MR imaging as a potential diagnostic test for metabolic myopathies: Importance of variations in the T2 of muscle with exercise. Am J Roentegenol 1993;161(2): 347–351.
10. Schedel H, Reimers CD, Vogl T, Witt TN. Muscle edema in MR imaging of neuromuscular diseases. Acta Radiol 1995;36(3):228–232.
11. May DA, Disler DG, Jones EA, Balkinson AA, Manaster BJ. Abnormal signal intensity in skeletal muscle at MR imaging: Patterns, pearls and pitfalls. Radiographics 2000;20:S295–S315.
12. West GA, Haynor DR, Goodkin R, Tsuruda JS, Bronstein AD, Kraft G, Winter T, Kliot M. Magnetic resonance imaging signal changes in denervated muscles after peripheral nerve injury. Neurosurgery 1994;35(6): 1077–1086.
13. Richardson ML, Zink-Brody GC, Patten RM, Koh WJ, Conrad EU. MR characterization of post-radiation soft tissue oedema. Skeletal Radiol 1996;25:537–543.
14. Utemi M, Hayashi K, Matsunaga N, Imamura K, Ito N. Denervated skeletal muscle: MR imaging. Work in progress. Radiology 1993;189(2):511–515.
15. Biondetti PR, Ehman RL. Soft-tissue sarcomas: Use of textural patterns as a diagnosis feature in postoperative MR imaging. Radiology 1992;183:845–848.

